# Evaluation of rotary file system (Kedo-S-Square) in root canal preparation of primary anterior teeth using cone beam computed tomography (CBCT)-in vitro study

**DOI:** 10.1186/s12903-021-02021-0

**Published:** 2022-01-18

**Authors:** Rasha H. Mohamed, Amina M. Abdelrahman, Aly A. Sharaf

**Affiliations:** 1 BDS, Faculty of Dentistry, Alexandria University, Alexandria, Egypt; 2 Professor of Pediatric Dentistry, Department of Pediatric Dentistry and Dental Public Health, Faculty of Dentistry, Alexandria University, Alexandria, Egypt

**Keywords:** Kedo-S-Square rotary file, Hand K-files, Hand H-files, Primary teeth, Pulpectomy, Cone-beam computed tomography

## Abstract

**Background:**

In recent years, pediatric endodontics has witnessed various advances including use of rotary files in pulpectomy. This study aimed to comparatively evaluate taper, amount of dentin removal and instrumentation time of the pediatric rotary Kedo-S Square file, hand K-files and H-files in primary canines using cone beam computed tomography (CBCT).

**Methods:**

60 primary canines were randomly assigned into three groups; A1 Kedo-S-Square rotary file (group I), hand stainless steel K file (group II) and hand stainless steel H file (group III). Teeth were mounted in vinyl poly siloxane impression material templates to be scanned before and after instrumentation by CBCT scans using Ondemand 3D software. Shaping ability of the files were evaluated in terms of taper of the canal and amount of dentin remaining of each group. Instrumentation time was recorded using a digital stopwatch.

**Results:**

Kedo-S Square removed a significantly less amount of dentin in both apical (P < 0.002) and coronal thirds (P < 0.014). Taper of the preparations showed significant differences as Kedo-S Square file showed good taper in maximum number of root canals, while maual K- and H-files showed poor taper in maximum number of root canals (P < 0.0001). Rotary Kedo-S Square files required less instrumentation time (P < 0.0001).

**Conclusion:**

The use of rotary Kedo-S Square files resulted in better conservation of tooth structure, superior tapering ability and least instrumentation time compared to hand K- and H-files.

## Background

Endodontic root canal preparation in primary teeth is considered to be a challenging procedure because of the perplexing anatomy, tortuous course of the canals, dynamic alteration at the root apex, close proximity to the succedaneous tooth bud as well as the perceived difficulties in behavioral management [1]. Pulpectomy is considered the treatment of choice for pulpally involved primary teeth in which the pulpal tissue is infected due to caries or trauma [2].

Biomechanical preparation is one of the most crucial phases of pulpectomy in primary teeth, which are targeted primarily during debridement of the canals [3]. The standardized method for cleaning and shaping of primary teeth was employed using hand files. Despite being commonly used as the most standard and widely accepted method for biomechanical preparation in primary teeth, certain limitations were associated with hand files as being time-consuming and the occurrence of iatrogenic errors such as lateral perforations, zipping, apical blockage, and canal transportation [4].

To overcome these limitations, Ni–Ti rotary files were first introduced in pediatric endodontics by Barr et al. in 2000 through use of Profile 0.04 taper rotary instruments in pulpectomy of primary teeth. It was found to be an efficient technique to debride the uneven walls of primary root canals and produce a uniform root canal shape that ended with a predictable obturation [5]. Crespo et al. concluded that usage of rotary files in deciduous teeth were more efficient in both root canal shaping and preparation time, facilitating higher quality of obturation of the root canal [6].

Rotary instrumentation performed in primary teeth was through use of rotary files designed specifically for permanent teeth until 2016. The taper and length were limitations in using existing rotary systems for permanent teeth when used in primary teeth [7]. This led to the occurrence of lateral perforations in the root surface especially in primary curved rooted canals [8]. This resulted in a great need for the design of an exclusive pediatric rotary file system that could be used in primary teeth [9].

The launch of new exclusive pediatric rotary files into the field of pediatric dentistry, has dramatically transformed the pediatric endodontic field [10]. Kedo files are rotary endodontic files, which are indigenously manufactured and designed for primary teeth in 2016. Kedo Dental is the name of the manufacturer, and the files were produced in Chennai, Tamil Nadu, India. Kedo rotary files include five generations of products, which were launched and are known respectively as [11]: Kedo-S rotary file, Kedo-SG, Kedo-SG blue [12], Kedo-S Square and Kedo-S Plus [13].

Kedo-S Square rotary file has revolutionized the arena of pediatric endodontics as being the first exclusive single pediatric rotary file system. It is the fourth generation of rotary Kedo-S files, which were introduced in 2019. It consists of two files, one file to be used for anterior primary teeth (A1) and one file to be used for posterior primary teeth (P1). It has a dual core made of NiTi heat-treated alloy and coated with titanium—oxide. The A1 Kedo-S Square file is used for preparation of primary incisors and canines. Kedo-S Square rotary files have a unique feature that includes a variably variable taper design that provides the flexibility and efficiency to facilitate consistently successful cleaning and shaping [10].

The two primary reasons of failure of endodontic rotary instrument, as proven by Sattapan et al. [14], are an excessive torsional and/or flexural load that generates stresses that exceed the elastic deformation capacity of the instrument, causing it to first deform plastically and then fracture. When the tip of the instrument binds in the root canal while the shank continues to rotate, torsion fatigue results [15]. Many variables impact the resistance of rotary instruments to torsional loads, including those linked to clinical use and those connected to the manufacturing process, in addition to the influence of flexural stresses [16]. Clinical use of files include the technique of instrumentation, type of motion used and extension of the access cavity5.features of the files itself are related to the manufacturing process such as type of alloy, heat treatment, surface treatment, cross sectional design, shaft length, tip size, pitch and taper [17–20].

Numerous methods have been used by researchers to assess the shaping ability of endodontic instruments including radiography, histological sectioning, electron microscopy, stereomicroscopy, computed tomography (CT), cone-beam CT (CBCT) and micro CT. CBCT is a novel noninvasive 3D digital imaging approach, which was used in this study to avoid the reported shortcomings associated with sectioning techniques and two-dimensional radiography [21].

No published scientific studies have evaluated the shaping effectiveness of the recently introduced Kedo-S Square rotary file. Therefore, the aim of the present study was to assess the shaping ability of the Kedo-S Square file in root canal preparation of primary canines in comparison to conventional hand K- and H-files through evaluation of the taper of the canals, residual dentin thickness and assessment of instrumentation time using CBCT.

## Material and methods

This in vitro study was conducted in the faculty of Dentistry, Alexandria University, Pediatric Department of Alexandria University and in a Dental Imaging Center, Alexandria. Ethical approval for the study was obtained from the Dental Research Ethics Committee, Faculty of Dentistry, Alexandria University, before the start of the study (International Number IORG 0008839, and Ethics Committee Number 0073-09/2019).

### Selection and storage of teeth

Sixty primary canines that were serially extracted or retained beyond the age of exfoliation were enrolled in this study. Length of the roots of the canines ranged from 7 to 10 mm or at least two-thirds of the roots were present [22]. Teeth, which had internal or extensive pathological root resorption or had undergone pulpectomy were excluded from the study. The selected teeth were washed and cleaned under running water to remove soft tissue debris. They were stored in sterile distilled water at room temperature until experiments were conducted.

### Sample size calculation and randomization

Sample size was estimated based on the following assumptions: confidence level = 95% and study power = 80%. The mean root canal taper difference of the middle third before and after root canal preparation using Kedo-S Square file = 0.1418 mm, while it was 0.0338 mm in the hand K-files group, with pooled standard deviation (SD) = 0.11113 [22]. H-files were assumed to have a similar effect to the K-files but different from the rotary systems used in pediatric endodontics [23]. Sample size was based on the difference between the Kedo-S-Square rotary file system and Hand K-files using the highest SD to account the variability. The minimum sample size was calculated to be 18 teeth per group, and this was increased to 20 to make up for laboratory processing errors.

Randomization was done using Random Allocation Software. Sixty teeth were randomized with equal allocation ratio of 1:1:1 and block size of six. A trial independent individual prepared a computer-generated randomization list, that was kept in an opaque sealed envelope, to allocate teeth that comply with the inclusion criteria to one of three arms.

### Grouping

Teeth were allocated to one of the three groups as follows: Group 1 (Rotary A1 Kedo-S Square), Group 2 (hand K-file) and Group 3 (hand H-file).

### Preparation of study specimens

The samples in all the groups were mounted in vinyl-polysiloxane impression material (Express™ XT Putty Quick, 3 M/ ESPE, Germany) in 12 acrylic templates. The custom-made acrylic templates were constructed with dimensions less than the field of view (FOV) of the CBCT machine. In order to maintain uniformity in all samples the teeth were placed in the labio-lingual direction where the mesial surface faced the template [24]. Pre-operative images of all the included primary canines were taken using CBCT. The type of CBCT was Sordex Scanora 3DX, made in Finland with 5 × 5 field of view. The software used was OnDemand 3D software (Seoul, Korea). It has the following features: version 1.0, build 1.0.10.7510, × 64 Edition, copyright (c) 2004–2017 Cybermed, and license key 1135113166 [25]. The exposure time was 6 s with an exposure dose 90 kV and 10 mA. The mode of measurements of the cervical, middle and apical thirds were standardized for all teeth specimens [22]. The standardizations were as follows: apical third of the canal was measured at 3 mm from the apex, middle third of the canal was measured at 6 mm from the apex and coronal third of the root canal was measured at 9 mm from the apex.

All the procedures for the three groups were done by a single calibrated operator. Intra-examiner reliability was determined for the six samples used for the pilot study where ICC values for the dentin thickness showed excellent agreement (ICC = 0.948). The mesio-distal tapering had ICC values of 0.780, which indicates good reliability.

All the steps needed to overcome any sort of bias were taken into consideration to standardize the parameters between the three groups. A No. 4 round carbide bur (Komet, Germany) was used on a high-speed hand piece under water cooling to remove the enamel and dentin layer or superficial caries if present. The endodontic access opening was prepared using No.330 pear shaped bur (Komet, Germany). Then No.10 size K file was used to determine the patency of the canals. The working length was kept 1 mm short of the apical foramen.

Group 1: The root canals were instrumented with the Rotary A1 Kedo-S Square file system (Reeganz Dental Care Pvt. Ltd. India) till the entire working length was covered in a lateral brushing motion 1–2 times in each tooth with X-Smart endodontic motor in a clockwise rotation motion (Dentsply, Wave one, Germany) at 300 rpm and 2.2 N cm torque. The A1 file is color coded with green and black bands on the handle with a tip diameter of 0.38 mm [10].

The rotary instrument was preceded by hand instrument K-file size No 25 and no pre-flaring was required. In order to maintain uniformity during canal instrumentation each Kedo-S Square file was used on up to 5 teeth, although it could be used on up to 12 teeth (nearly 36 canals) as per the manufacturer’s recommendation [26].

Group 2: The root canals were manually prepared with 21 mm stainless steel K-files 0.02 taper from size #15 and the final size was #40 (Dentsply Maillefer, Ballaigues, Switzerland) using the quarter turn and pull technique.

Group 3: The root canals were manually prepared with 21 mm Stainless steel H-files from size #15 and the final size was #40 (Dentsply Maillefer, Ballaigues, Switzerland) using retraction motion (push and pull) technique.

Each hand file was used on up to five teeth and then changed in order to maintain uniformity during root canal instrumentation [27]. Copious irrigation solution was used in order to totally eliminate necrotic pulpal tissue. This irrigation was standardized to 10 ml of 1% sodium hypochlorite, followed by saline in all groups after each file use during the entire cleaning and shaping procedure. The files were lubricated with Ethylenediaminetetraacetic acid (EDTA gel)(RC-Prep, Premier Dental Products, USA) every time during in biomechanical preparation to avoid instrument fracture and separator deformation [28]. Instrumentation time was recorded in (minutes-seconds) by a trained dental assistant using a digital stopwatch. Instrumentation time is the amount of time needed negotiate and shape the canals after access and determination of working length were done [29].

### Scanning procedure

The samples in all the groups were mounted again in the templates in a similar manner and were subjected to CBCT scanning as done preoperatively [27]. Post instrumentation (CBCT) scans were conducted to analyze the internal three-dimensional root canal shapes in the three groups. OnDemand 3D software was used to get sagittal and axial cuts for all the imaged teeth. Standardized measurements were taken to measure the coronal, middle and apical thirds.

### Assessment of shaping ability

Shaping ability of the files was evaluated by measurements of the residual dentin thickness from the axial cut of the CBCT in mesial, distal, labial and lingual directions at three different levels, coronal third, middle third, and apical third. Taper of the canal was measured from the sagittal cut of the CBCT in the three predefined positions in the mesio-distal direction [22, 30].

According to Gambill JM et al. [31], the percentage of dentin removed was calculated as (X1 − X2) or (Y1 − Y2), multiplied by 100% and divided by X1 or Y1, depending on the side (X or Y), where X1 is the shortest distance from the outside of the root to the periphery of the non-instrumented canal; X2 is a corresponding similar distance in the respective instrumented root canal; Y1 is the shortest distance from the inside of the root to the periphery of the non-instrumented canal; and Y2 is a corresponding similar distance in the respective instrumented root canal.

Taper of the canals, in the mesio-distal direction, was measured in the three defined levels using the OnDemand software [22, 32].

(i) Good taper was identified when there was a progressive reduction from coronal, middle to apical third of the root canal [33, 34].
(ii) Poor taper was identified when either there was same or increased readings from coronal, middle to apical third of the root canal [33, 34].

### Statistical analysis

Normality was checked using descriptive statistics, plots, and normality tests (Shapiro Wilk test). Means and standard deviations (SD) were calculated for normally distributed variables (Dentin thickness, Mesio-Distal taper and instrumentation duration), in addition to median and interquartile range (IQR) for not normally distributed variables (Percent change).

Taper ability was presented as frequency and percentage as it was dichotomized as: good tapered preparation where the taper of the canal after instrumentation showed a progressive reduction from coronal, middle to apical third of the root canal, and poor tapered preparation where there were either similar or increase in the readings from coronal, middle to apical third of the root canal after instrumentation [32].

Percent change for all variables was calculated according to the following formula: [(values after instrumentation—values before instrumentation)/values before instrumentation) × 100]. Significance level was set at p value < 0.05. Data were analyzed using SPSS for Windows version 25.0.

## Results

**Fig. 1:**
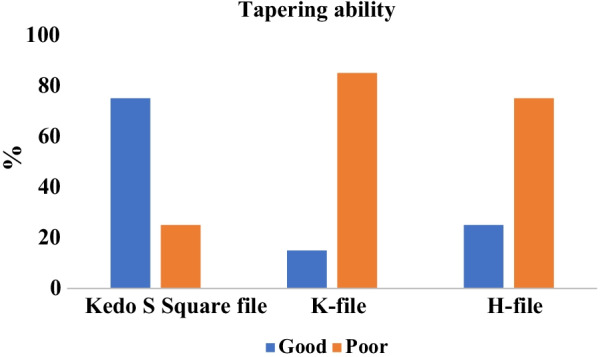
Tapering ability of Kedo-S Square file, K- and H-files

**Fig. 2:**
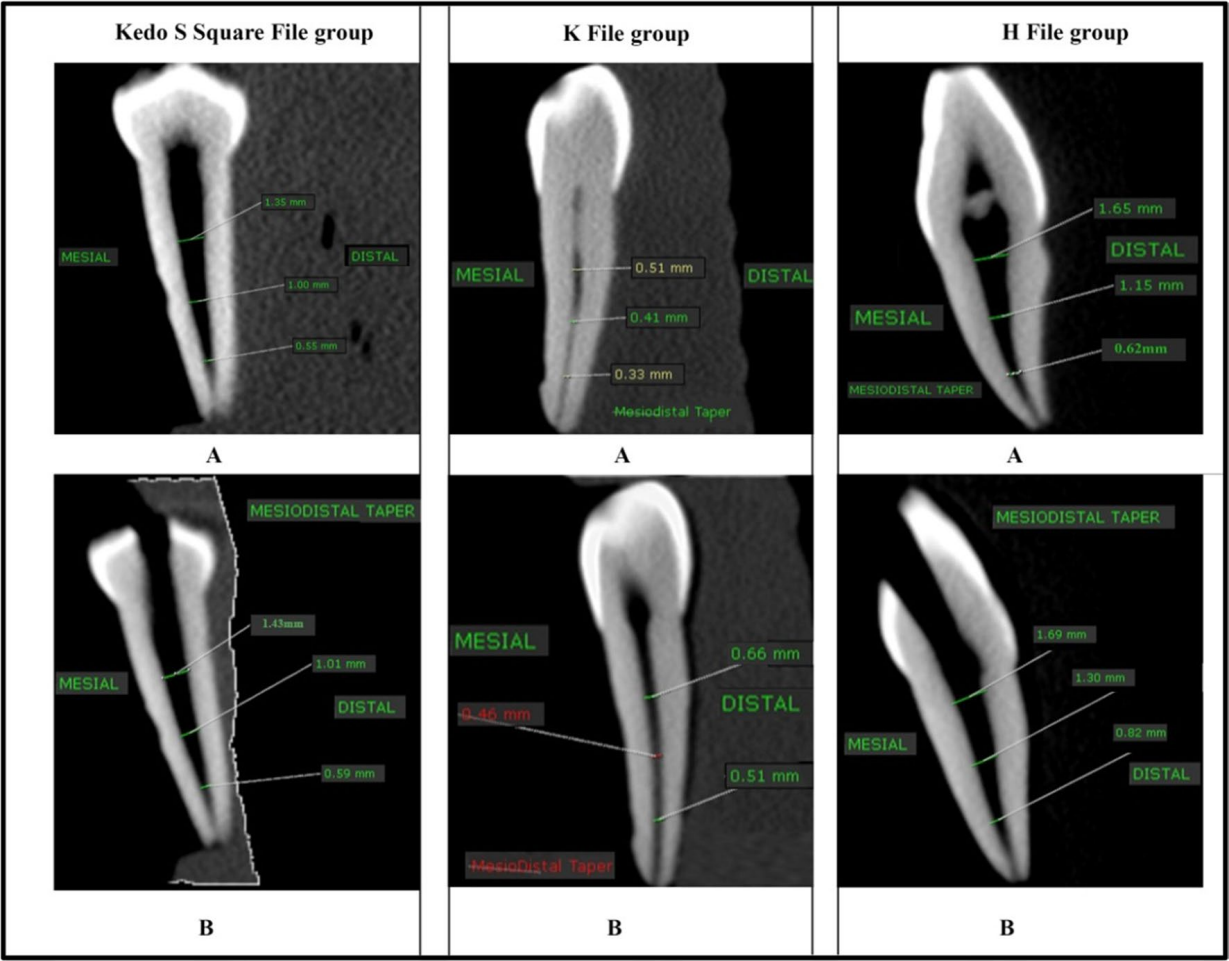
Pictorial representation showing sagittal cut of CBCT: **a** Pre- and Post-preparation CBCT scan images showing good taper with Kedo-S Square rotary file. **b** Pre- and Post-preparation CBCT scan images showing poor taper with manual K and H files

Regarding the tapering ability, the canal tapers prepared with rotary Kedo-S Square file were more conical compared to the H-and K-files and were significantly different (P < 0.0001) as demonstrated in Fig. 1. The shaping ability was not significantly different amongst the hand files. Taper of the canal preparation was significantly better using the Kedo-S Square group file, with a larger number of good tapered preparation 15 (75%) compared with both H-files 5 (25%) and K-files groups 3 (15%), respectively as shown in Fig. 2.

**Fig. 3:**
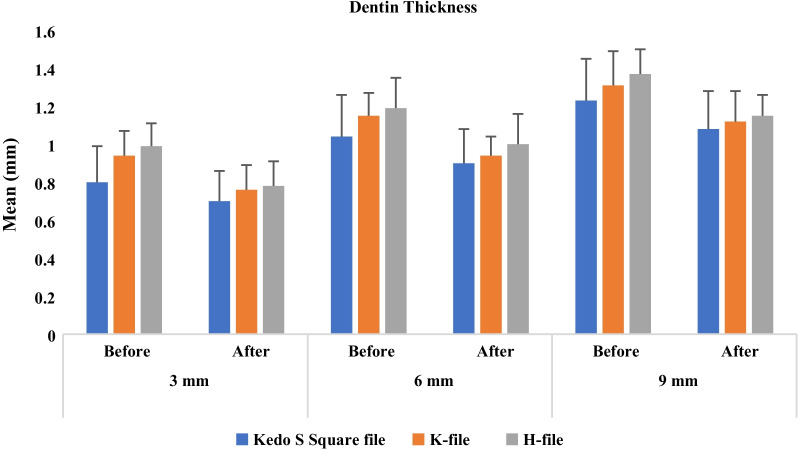
Mean residual dentin thickness (mm) following use of Kedo-S Square file, K- and H-files before and after instrumentation at different levels

The dentin thickness before and after instrumentation is presented in Fig. 3. Comparison of the dentin thickness before and after instrumentation showed significant reduction at all three levels in all groups (P < 0.0001). However, no statistically significant difference in dentin thickness was found between the three groups after instrumentation (P > 0.05).

**Fig. 4:**
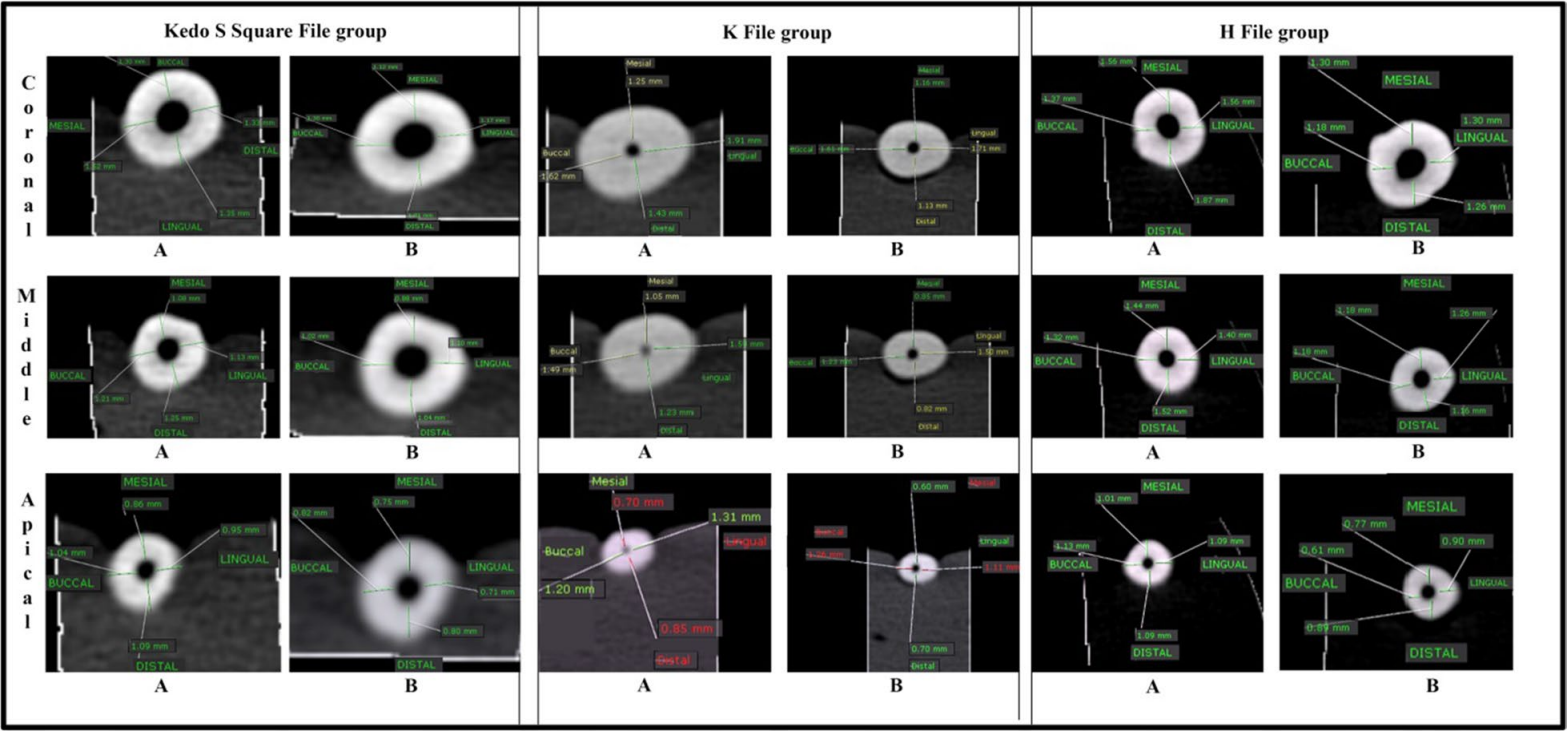
Pictorial representation of axial cut of CBCT for Kedo-S Square, K- and H-file groups: **a** Pre-operative CBCT image of amount of dentin thickness at apical, middle and coronal thirds, **b** Post-operative CBCT image of amount of dentin thickness

Regarding the median amount of dentin removed, a significant difference was found between the Kedo-S Square file and H- and K-files at the apical level (P = 0.002), and between Kedo file and H-file at the coronal third (P = 0.014). The Kedo-S Square file removed the least amount of dentin (14.02%, 12.75%, 10.84% at the apical, middle, and coronal third, respectively) followed by the K-file group (19.77%, 15.71%, and 13.85%, respectively) and it was the highest in H-file group (21.51%, 16.34%, and 16.90%, respectively) as shown in Table 1/Fig. 4.

**Table 1:** Comparison of percent reduction in Dentin thickness among three files at different levels

Percent reduction (mm)		Kedo-S square file (n = 20)	K-file (n = 20)	H-file (n = 20)	H test (*P* value)
3 mm	Mean ± SD	12.79 (6.60)	19.09 (6.70)	20.92 (6.85)	12.843 (0.002*)
	Median (IQR)	14.02 (12.80)^a^	19.77 (9.42)^b^	21.51 (8.88)^b^	
6 mm	Mean ± SD	13.41 (7.11)	17.76 (8.79)	16.60 (4.09)	5.120 (0.077)
	Median (IQR)	12.75 (8.65)	15.71 (4.90)	16.34 (4.49)	
9 mm	Mean ± SD	11.96 (7.27)	14.46 (5.62)	16.31 (4.19)	8.554 (0.014*)
	Median (IQR)	10.84 (9.78)^a^	13.85 (7.56)^ab^	16.90 (6.41)^b^	

H test: Kruskal–Wallis test

*Statistically significant different at *p* value ≤ 0.05

^a,b^Different letters denote significant difference between groups

Instrumentation time elicited with Group 3 H-file (7.67 (2.14) min) was significantly longer than Group 2 K file (5.81 (3.30) min) and was least in Group 1 rotary Kedo-S Square file (2.12 (0.82) min) with (P < 0.0001).

## Discussion

Mechanical cleaning of root canals can be done using endodontic broaches, hand files, reamers and nickel‑titanium (Ni–Ti) rotary files. In the field of pediatric endodontics, conventional hand instrumentation has been utilized for decades and is regarded as the gold standard method. Because manual techniques are time-consuming, they frequently cause fatigue in both the operator and the child, which has a significant impact on the aspect of behaviour management in pediatric dentistry. Usage of hand files in preparation of curved canals frequently causes undesirable aberrations such as elbows, zips and danger zones because of their inherent stiffness [35]. Consequently, usage of stainless‑steel files in narrow curved canals is considered to be difficult as apical enlargement is limited, hindering good quality obturation [36].

From the merits of rotary instrumentation are having greater apical enlargement capacity, reducing apical transportation and improving root canal shape over conventional hand files [8]. In deciduous teeth, a larger amount of instrument separation was seen while using permanent rotary systems [34]. In 2017, a survey showed that 66% of dentists needed an exclusive pediatric rotary file for faster preparation and better accessibility [7]. Thus, as recommended by Kuo et al. [37] to modify these files and to design new NiTi rotary files exclusively for primary teeth to accommodate the ribbon shaped canals of primary teeth [4].

The hand 2% tapered files mean that every 1 mm of the file, the taper increases by 0.02 mm. Consequently, the file with a 4% taper will result in better preparation than that of a 2% tapered file. This indicates that the entire length of the file will engage the whole root canal wall, requiring several numbers of files to complete the preparation [38, 39]. The concept of variable tapering root canal preparation was established at Buchanan in 2000, in which the root canal width gradually increases towards the coronal third [40]. Variable-tapered files have different tapers of increasing and decreasing angles along that portion of the file. The variable-tapered file was better at preserving tooth structure than fixed-tapered endodontic files in the mid-root curvature [41].

Regarding tapering ability, this study showed that there was a significant difference in taper of root canal preparation between the three groups as 75% of the Kedo-S Square file preparation, 5% of H-file preparation and 3% of the K-file preparations were identified as good tapered preparations. This can be attributed to the design of the Kedo-S Square files as it has dual cross section and a variable taper corresponding to the anatomy of root canal of primary teeth when compared to the fixed narrow taper hand files. The taper of A1 is 6 to 8%, as the first 5 mm of the file is of 6% taper followed by increase in taper by 7 and 8%. This results in higher cervical enlargement and restricted apical preparation that prevents extrusion of the obturating material [42, 43].

Moreover, its well-designed working length (17 mm length of the file and 13 mm length of the cutting edge) and tip diameter 0.38 mm, avoids the occurrence of lateral perforation at the apical region. The hand K- and H files lack the above-mentioned properties necessary for efficient preparation of canals, which results in reduced number of good taper preparations using the conventional hand files [44].

The dual cross section of Kedo-S Square means that the apical 5 mm of the file has a triangular cross section whereas the rest of the file at the coronal region has a tear drop cross section. This feature enhances tapering toward the apex of the canal. This will create less apical preparation that prevents lateral strip perforation of the canals and more coronal preparation of the root canals, which will permit easy flow of the obturating material into the canals of primary teeth. The tear drop cross section can also be seen in the cross section of hand H-file, which allows for easier pulling capacity motion of the pulp from the canals during instrumentation [9]. H-files have a tear drop shaped cross section and 2% taper that does not provide more conical enlargement [45]. It was also noticed that the hand K- and H-files created irregular non-uniform poorly prepared canals and have limited coronal preparation, which hinders optimal flow of the obturation material [1].

These results resemble the findings of Srinivas et al. [22] who concluded that Kedo-S rotary files creates more conical and good tapered canal preparation when compared to hand K-files. Nahid Razani [33] reported that CBCT assessments showed that better tapered preparations were obtained using M-two rotary files than the hand K-files. No significant difference was found in taper in a study done by Seema et al. [30] wherein they compared hand K files, rotary Prosper files, and rotary Kedo-S files using CBCT. However, 85.7% of rotary ProTaper and 80.95% of Kedo-S preparations showed better taper, but only 71.4% of hand preparations were good preparations.

Sufficient residual dentin thickness is essential to provide enough resistance to lateral and occlusal forces for an endodontically treated tooth. The aggressiveness of the root canal instrument and dentin removal are positively correlated [46]. In this study, Kedo-S Square file seemed to result in more conservative and meticulous removal of dentin. This is desirable to preserve the integrity of thin-walled primary root canals. The percentage of dentin removal was significantly highest with H-file and least in Kedo-S Square file in both apical and coronal thirds.

In the middle third, no significant difference was found between the three groups although hand files had also removed more dentin than the rotary Kedo-S Square. This could be attributed to the increased straightening of the canal by less flexible hand K and H files when compared to more flexible M-wire technology of Kedo S Square files which is in accordance with the findings of Radhika et al. [47] M-wire alloys are produced by a series of heat treatment and annealing cycles which provides them superior strength [48]. In case of having 2 files with same cross section, M-wire alloy resists fracture better than conventional alloy [49]. The benefit of the M wire technology is that these instruments exhibit greater resistance to cyclic fatigue and render the instrument more super elastic than conventional NiTi instrument [50].

The increased flexibility of the Kedo S Square file as a result of being heat treated helps in adaptation of the file to the primary canal curvature, unlike K-files that lead to more transportation and zipping [1, 10]. Heat treatment of Nickel Titanium files enables the files to attain higher torsional resistance when compared to conventional files [51]. According to Tabassum et al., the extreme ductility offered by various heat treatments may explain the superior flexibility and hence increased resistance to failure of these instruments [52].

Hence, a greater amount of dentin was removed by hand files than by rotary Kedo S Square file,which could be attributed to the non-cutting safety tip of the Kedo S Square files and to the indiscriminate and aggressive cutting action of stainless-steel files [36]. H type instrument has better cutting efficiency than K type instruments because H file has a positive rake angle where its blade cuts rather than scarps dentin unlike the K file having a negative rake angle [53].

The variable taper of the rotary Kedo-S Square file creates conservative coronal shape, which preserves dentin with deep apical shaping enabling better access for irrigation and cleaning resulting in three-dimensional obturation [26]. The principle behind varying taper is that each successive file is only engages a minimal aspect of the canal wall [54].

This is in accordance with the results of Seema et al. [30] wherein no significant difference between hand K and rotary Kedo-S files with respect to the amount of dentin removed at the middle and apical thirds; however, at the coronal third, the hand K removed significantly more dentin than the Kedo-S file in the axial cut of CBCT. Musale et al. [46] conducted a comparative assessment of dentin removal and showed that at all three levels, the K-file removed more dentin than Hero Shaper Classic with a taper of 0.04 mm, and the rotary files removed 18% less dentin in second primary molars and 17% less in first primary molars. Barasuol et al. [55] K file led to more dentin removal in the apical third than ProDesign Logic and Reciproc files as assessed using micro computed tomography.

On the contrary, other studies have reported that rotary files removed greater amount of dentin than hand files as a result of using larger tapered rotary files, which removed more dentin than the constant 2% hand files [56, 57]. Canoglu et al. [58] concluded that there were no significant differences with regards to dentin removal between the three preparation techniques (Profile 0.04 ISO), ultrasonic (K-Type/Satelec), and stainless-steel hand file K-file.

Reduced instrumentation time is highly critical in influencing the behavior of children and their co-operation in the dental chair, therefore reducing the operator’s and child’s fatigue[32]. In the current study, a significantly decreased instrumentation time was recorded with Kedo-S Square rotary file ( 2.12 min) when compared to the K-file (5.81 min) and H file (7.67 min), which is in accordance with randomized controlled study done by Lakshmanan et al. [59], where they compared Kedo-S Square rotary file to hand K and H hand files and found that Kedo-S Square rotary file had the least instrumentation time (73.4 s) when compared to the K-file (105.6 s) and highest in H file (126.8 s). Panchal et al., reported reduced instrumentation time and better obturation quality with rotary Kedo-S system than the hand K and H files groups that positively affects the cooperation of the children [28].

It must be kept in mind that the present study was conducted using only single rooted primary canines with straight roots and no apical resorption. These results were obtained in a laboratory setting and results could be different in a clinical setting. Hence, use of pediatric rotary files is recommended over hand files in order to attain successful pulpectomy technique in a simple and quick manner with proper debridement of root canals of primary teeth [27, 43].

There are some factors that enabled a reduced instrumentation time with Kedo-S-Square compared to hand K and H files. Firstly, lesser number of files in the case of Kedo-S Square (i.e., 1 single file) are used in each canal for efficient cleaning and shaping compared to the use of size No. 15 to No. 40 hand K and H files (i.e., six) for effective canal preparation. Hand files have constant taper resulting in the need for use of more files to complete the preparation [60]. Secondly, the dual cross section of the Kedo-S Square file (tear drop cross section), which is similar to H file, results in easier and complete extirpation of pulpal tissue, as opposed to the need to sequentially use of increased sizes of hand K and H-files to accomplish this task.

However, contrary to our results, Madan et al. [61] and Katge et al. [62] concluded that there was an increase in root canal instrumentation time with the use of the rotary file system in primary teeth, which may be related to the operator’s knowledge level, skill level, and experience with rotary endodontics [27].

In this study, Kedo-S Square pediatric rotary file was evaluated to understand if the newly designed exclusive rotary files for primary teeth, with a modified variable taper and shorter length, could be an alternative to the existing manual files. Further studies to evaluate patients’ acceptance of the Kedo-S Square and Kedo-S Plus rotary files are needed. Moreover, long-term clinical and radiographic success rates should be performed to reach a precise conclusion.

## Conclusion

Within the experimental conditions of the present study, the following conclusions can be drawn:

- Use of rotary Kedo-S Square resulted in good taper in maximum number of root canals unlike hand K and H files, which gave rise to maximum number of poor tapered preparations.
- Use of Kedo-S Square files resulted in significantly greater conservation of tooth structure compared to hand K and H files at all levels except at the middle third, where there were no significant differences between the three groups.
- Kedo-S Square file system required significantly less instrumentation time than the hand K and H file systems.

## Data Availability

The data that support the findings of this study are available from the corresponding author upon reasonable request.

## Abbreviation

CBCT: Cone beam computed tomography
